# Rapid Determination of Total Tryptophan in Yoghurt by Ultra High Performance Liquid Chromatography with Fluorescence Detection

**DOI:** 10.3390/molecules25215025

**Published:** 2020-10-29

**Authors:** Mena Ritota, Pamela Manzi

**Affiliations:** CREA-Centro di Ricerca Alimenti e Nutrizione, Via Ardeatina 546, 00178 Rome, Italy; mena.ritota@crea.gov.it

**Keywords:** tryptophan, ultra high performance liquid chromatography, yoghurt

## Abstract

Tryptophan (TRP) is an essential amino acid which cannot be synthesized by humans and animals, but has to be supplied by exogenous sources, notably through the diet. The bulk of dietary TRP flows into the synthesis of body’s proteins, but the TRP metabolism also involves several biochemical reactions (i.e., serotonin and kynurenine pathways). Defects in the TRP transport mechanism or catabolism are related to a large number of clinical abnormalities. Therefore, dietary TRP intake is necessary not only for the body’s growth but also for most of the body’s metabolic functions. Among protein-based foods, milk proteins provide a relatively high amount of TRP. In this paper, a rapid chromatographic method for TRP determination in yoghurt, by ultra high performance liquid chromatography on a reversed-phase column with fluorescence detection (280 nm Ex; 360 nm Em), is provided. A linear gradient elution of acetonitrile in water allowed TRP analysis in 8.0 min. The limit of detection and limit of quantification of the method were 0.011 ng/µL and 0.029 ng/µL, respectively, using 5-methyl-l-tryptophan as the internal standard. The analytical method was successfully applied to commercial yoghurts from different animal species, and the TRP values ranged between 35.19 and 121.97 mg/100 g (goat and cow Greek type yoghurt, respectively).

## 1. Introduction

Tryptophan (TRP) is an essential amino acid needed for normal growth, and is involved in the synthesis of different bioactive compounds, such as nicotinamide (vitamin B6), melatonin, tryptamine, kynurenine, 3-hydroxykynurenine, and quinolinic and xanthurenic acids [1].

In lower organisms, TRP is formed through the condensation of serine with an indole group by the action of tryptophan synthase [2]. In humans and animals, though, TRP cannot be synthesized because they are lacking in tryptophan synthase [2]. Therefore, TRP has to be supplied to the body by exogenous sources, especially through the diet [2]. Besides participating in the formation of the body’s proteins, TRP is involved in numerous chemical reactions. 

TRP and its metabolites seem to have the potential to contribute to the therapy of autism, multiple sclerosis, cardiovascular, chronic kidney and inflammatory bowel diseases, cognitive function, depression, and microbial infections [1]. TRP transport through the cell membranes is competitively inhibited by the other large neutral amino acids (NAA), such as valine, leucine, isoleucine, phenylalanine and tyrosine [3]. 

From these premises, it is clear that TRP intake through the diet, or the intake of TRP-rich proteins, is necessary not only for the body’s growth, but also to carry out most of the body’s metabolic functions. However, the wide use of TRP as a dietary supplement for its potential health benefits has raised the issue of assessing its safety [1], so much so that an upper limit of safe intake for diet-added tryptophan of 4.5 g/day has been proposed in young adults [4].

Among food proteins introduced through the diet, cereals, and especially maize, are generally poor in tryptophan content; as such, TRP may represent the nutritionally limiting amino acid in these food items [5]. Egg, soy, beans, seafood and poultry proteins, instead, have been described as good sources of tryptophan [6].

Milk proteins are particularly rich in TRP, which can be released by the proteolytic enzymes either as a free amino acid or as a part of small peptides with functional activities [2]. Furthermore, it has been shown that TRP released during in vitro gastrointestinal digestion is one of the main factors responsible for the radical scavenging activity of digested bovine milk [7]. TRP intake through the diet considerably varies depending on the food protein type, since TRP distribution can significantly differ among the various fractions [8]; in cow’s milk proteins, for example, α-lactalbumin contains around 6% TRP, whereas bovine serum albumin and β-casein are extremely poor in TRP [9]. Furthermore, the TRP bioavailability through dietary intake might be reduced during food processing or cooking, mainly by oxidative degradation or cross-linking among proteins [10,11], as well as by decarboxylation reactions [12].

The nutritional and safety aspects of tryptophan emphasize the need for reliable analytical methods for its determination in food. The first rapid method for tryptophan determination dates back to 1970, when Gaitonde and Dovey [13] proposed a colorimetric method, whereby TRP reacted with acid ninhydrin giving a yellow product, which was spectrophotometrically revealed at λ = 390 nm. However, this method needed to be corrected for tyrosine absorption [14]. Later, Inglis [15] developed a method for amino acid determination by preventing tryptophan degradation, which generally occurs in the acid conditions of protein hydrolysis (HCl 6 N at 110 °C for 22 or 24 h [16]), by protecting TRP with tryptamine. Yamada et al. [17] improved this method by modifying the proteins with vapor-phase S-pyridylethylation before the hydrolysis, and then treated the modified proteins with mercaptoethanesulfonic acid at 176 °C. However, all these early methods were applied to pure proteins, without considering the matrix effect.

When referring to a food product, one of the main concerns is certainly the release of the protein-bound tryptophan from the matrix. Acid hydrolysis generally carried out for the total determination of most amino acids cannot be performed for TRP analysis, because it is destroyed during acid hydrolysis [16]. Therefore, alkaline hydrolysis, as reported by Steven and Jorg as far back as 1989 [18], is currently the pre-treatment method of choice for tryptophan determination in foods.

TRP detection and quantification can be carried out with different analytical techniques. Near infrared spectroscopy (NIRS) was used to determine TRP, as well as other amino acids in dairy products, and in particular to evaluate cheese ripening [19]. Even if these spectroscopy methods offer the advantages of a short time of analysis and a poor sample preparation, they need huge amounts of samples for the calibration and validation of the models. Ion exchange chromatography (IEC) with fluorescence detection was also employed to determine TRP in pure proteins and feedstuffs [20], but IEC needed post-column derivatization with *o*-phthalaldehyde (OPA), thus resulting in further time- and chemical-consuming steps. The determination of tryptophan in infant formula by high performance liquid chromatography (HPLC) with UV detection (λ = 254 nm) needed a derivatization step, with phenylisothiocyanate (PITC), in addition to the protein hydrolysis prior to the HPLC analysis [21,22]. Derivatization was necessary due to the poor absorbance of TRP in the UV spectral region, but this could result in a time- and chemicals-consuming procedure. Furthermore, Tsopmo et al. [23], regardless of the use of the more sensitive tandem mass spectrometry (MS/MS) detector, derivatized TRP with PITC, in order to evaluate the TRP in human milk, infant plasma and peptide fraction.

Fluorimetric detectors could be employed to increase the selectivity and sensibility of an HPLC method [24]. Furthermore, TRP exhibits a strong native fluorescence [25], which allows one to avoid the derivatization generally needed for most amino acids determined by HPLC. 

Therefore, the aims of this paper were as follows: 1) to develop a robust, rapid and cost-effective method for tryptophan detection in yoghurt based on ultra high performance liquid chromatography (UHPLC) by means of a fluorescence detector; 2) to evaluate the levels of tryptophan in commercial yoghurts from the milk of different animal species. The analytical technique of choice in this study was UHPLC, since this chromatography has the advantages of speed, enhancing resolution and peak efficiency, as well as requiring smaller amounts of solvent compared to the traditional HPLC, so it can be used for fast and eco-friendly analysis. To the best of our knowledge, this is the first time that this technique has been applied for the analysis of TRP in yoghurt samples.

## 2. Results

### 2.1. Chromatographic Method Validation

The alkaline hydrolysis with NaOH, according to the method of Steven and Jorg [18], was carried out to extract TRP from the yoghurt samples. Partial disruption of TRP can occur during hydrolysis: these losses can be corrected based upon the recovery of an internal standard [16], and 5-methyl-l-tryptophan (M-TRP) has been revealed as the preferred internal standard for TRP analysis [26]. Furthermore, the presence of foreign substances in a matrix may cause a bias by increasing or decreasing the signal attributed to the measurand [27]. Due to the absence of a suitable reference material to estimate the potential influence of the interferences of the yoghurt matrix on the analysis of TRP (the so called “matrix effect”), the approach of recovery tests (using spiked samples) was used [27]. In the recovery value test, the original concentrations of TRP in the yogurt samples were determined using the calibration curve described below. Each of the yoghurt samples was then spiked, prior to the extraction, with a known concentration of TRP (at different levels, ranging from 0.061 to 0.152 µg/mL), and the total TRP concentrations of the spiked samples were calculated using the same calibration curve. The recovery values, adjusted for the value of M-TRP, ranged between 97.36% and 100.12%, with a mean value of 97.27% (Table 1).

The linearity range of the method was evaluated by injection, in triplicate, of the following TRP standard solutions: 1.105 µg/mL: 0.848 µg/mL; 0.424 µg/mL; 0.553 µg/mL 0.1696 µg/mL and 0.0848 µg/mL. The linearity range investigated covered the entire measurement range of the samples.

The calibration and quantification of TRP in the yogurt samples were obtained by the standard addition method. The calibration curve (y = 502654x) was obtained with a correlation factor R^2^ = 0.9995, with the error of curve equal to 1751. The limit of detection (LOD) and limit of quantification (LOQ) of the method were 0.011 ng/µL and 0.029 ng/µL, respectively.

The method’s precision was evaluated through repeatability and reproducibility measurements; the method resulted in a good precision, and the intra- and inter-day relative standard deviation (RSD %), on pure standards and on yoghurt samples, are shown in Table 2 and Table 3, respectively.

Furthermore, the relative standard deviation in retention times was less than 0.2% and 0.3%, for intra-day and inter-day, respectively.

**Figure 1:**
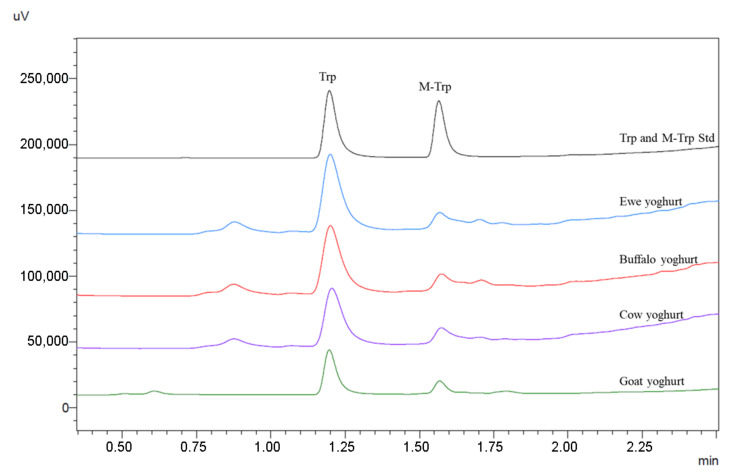
Chromatographic profile of a TRP and M-TRP standard solution and yoghurt samples extracts, according to the chromatographic conditions reported in Section 4.

The proposed chromatographic method allowed TRP determination in yoghurt samples in a relatively short time; the total chromatographic running time was 8.0 min, including column reconditioning, with a TRP retention time equal to 1.197 min and a 5-methyl-l-tryptophan (M-TRP) retention time equal to 1.564 min (Figure 1).

### 2.2. Tryptophan Levels in Commercial Yoghurts

The proposed method was applied to the analysis of the TRP contents in various commercial yoghurts obtained from the milk of different animal species. The results, reported in Table 4, showed a great variability.

Among the whole yoghurt samples, there were significant differences in the TRP levels (*P* < 0.05): ewe milk yoghurts showed the highest TRP concentration (on average 62.94 mg/100 g), while goat milk yoghurts had the lowest one (36.40 mg/100 g). Furthermore, whole cow yoghurt samples showed the greatest sample heterogeneity, with TRP levels ranging between 40.78 and 49.31 mg/100 g.

Among the cow yoghurts, the highest TRP content was observed in the Greek type samples (on average 121.47 mg/100 g), followed by lactose free and whole cow yoghurts (49.15 mg/100 g and 44.60 mg/100 g, respectively). The highest TRP value reported in the Greek type samples is justified by their high protein contents (about 9 g/100 g), due to the particular technological process whereby the liquid whey is removed, obtaining a thick and creamy product.

## 3. Discussion

### 3.1. Method Validation

Due to the presence of the indole ring in the tryptophan structure, which is degraded under the acid conditions generally used in the protein hydrolysis, TRP cannot be analyzed by the standard method for amino acid analysis [1]. Many attempts have been made over the years, from enzymatic to modified acid hydrolysis, but all these procedures suffer from incomplete TRP recovery [20,28]. Therefore, alkaline hydrolysis is the common procedure for TRP analysis in foods. Among the different alkalis used for protein hydrolysis [29], NaOH does not suffer for the inconvenience of solubility in water, unlike Ba(OH)_2_ and LiOH, and it avoids the precipitation/adsorption problems associated with the use of Ba(OH)_2_ [20]. Therefore in this study, an alkaline hydrolysis with NaOH 4.2 M, according to the method of Steven and Jorg [18], was performed.

While many scientific attempts have been made for TRP determinations in biological and pharmaceutical samples [30,31,32,33,34], as well as in non-dairy food items [25,35,36], very few works have been reported in the literature about TRP determinations in dairy products, and in particular in yoghurt.

The limit of detection (LOD) of this method (0.011 ng/µL) is comparable to that reported by Yılmaz and Gökmen (0.0165 ng/µL in yoghurt) [37], who even used a more sensitive detector (tandem mass spectrometry) to determine TRP and its derivatives in foods. Furthermore, our LOD is lower compared to that obtained by Liu and Xu for TRP analysis in milk [38], who employed a selective electrochemical sensor; the method proposed, in fact, showed a limit of detection of 6 μM, corresponding to 1.226 ng/µL. Only the methods proposed by Wang et al. [39], who developed a modified electrochemical sensor to determine TRP in milk, and by Baytak and Aslanoglu, who had to resort to a nanosensor for TRP determination in cow’s milk [40], showed lower LOD values (0.0035 ng/µL and 0.00135 ng/µL, respectively). Even though the electrochemical sensors generally allow a simple and rapid determination of the analytes in food matrices without any sample pretreatment, TRP analysis requires a modification procedure of the electrodes, since under the traditional working electrode conditions TRP oxidation suffers from high overpotential and sluggish kinetics [41]. These electrode modifications may result in chemical reagent consumption. Furthermore, electrochemical sensors are still little used in the laboratories for routine analysis.

The proposed chromatographic method allowed TRP determination in yoghurt samples in a relatively short time (total chromatographic running time = 8.0 min). This was possible thanks to the high speed of the UHPLC technique. Furthermore, Delgado-Andrade et al. [42] proposed a liquid chromatography-based method with fluorescence detection for TRP analysis in milk-based ingredients, but the total chromatographic time, even if not specified by the authors in the text, was at least 10 min. The running chromatographic time of this proposed method was even shorter than that proposed by La Cour et al. [43] with UHPLC–single quadrupole mass spectrometry, who reported a total analysis time of 11.5 min for TRP analysis in plant materials and dog foods, even if the author stated a possibility of shortening the running time.

The results reported in this study show that the proposed chromatographic method can be used for the routine analysis of TRP in foods; it is low in terms of time and chemical consumption, it ensures a low sensitivity, and the analytical equipment is easy to access and much less expensive than the more sensitive mass spectrometry.

### 3.2. Nutritional Evaluation of Eryptophan Levels in Commercial Yoghurts

Regarding the TRP levels in the yoghurt samples, the results obtained in this study are in agreement with those previously reported by Gambelli et al. [44] in commercial yoghurt samples, through a similar analytical method, but using an HPLC. The TRP levels reported in this study are also of the same order of magnitude as those reported by Posati and Orr [45] on similar commercial samples. Furthermore, Cañabate-Díaz et al. [46] evaluated the total TRP content in commercial yoghurts, but they reported slightly higher levels (374 mg/kg) by using a completely different method based on heavy atom-induced room temperature phosphorescence.

On the contrary, our results are very high compared to those reported by Bertazzo et al. [47] on similar food items (TRP = 0.7 µg/mL in yoghurt samples); the main difference is due to the fact that the authors [47] did not hydrolyze the commercial yoghurt samples, but carried out the HPLC analysis on the supernatants directly after centrifugation, so their results referred to the “free” TRP content of yoghurts. Furthermore, Yılmaz and Gökmen [48] reported similar results for free TRP in commercial yoghurts, with values ranging between 3.2 and 13.4 mg/kg dry weight. The differences in the free amino acid contents observed by the authors [47,48] could be due to the different microorganisms involved in the production of the yoghurts, since free amino acid content has been shown to be influenced by the interactions between the microorganisms involved in the yoghurt fermentation [48], and by the different strains of the microorganisms employed [49].

Differences in the total TRP levels of yoghurt samples from the milk of different animal species are essentially due to the different protein contents [44]. However, Yılmaz and Gökmen [37] observed the presence of TRP, together with its derivatives, in the kynurenine pathway in commercial yoghurts, suggesting a fermentation effect on the level of TRP and its derivatives. Similar findings were reported also by Bertazzo et al. [47], who observed TRP and its derivatives in both the serotonin and kynurenine pathways in milk and fermented dairy products. The authors also reported an increase in the free TRP levels with increasing fermentation, due to the proteolytic action of the added cultures, thus corroborating the hypothesis of Yılmaz and Gökmen [37]. 

The highest TRP content of yoghurt from ewe milk could be due to the higher general TRP content in ewe milk compared to milk from other animal species [50]. Similar values were reported for TRP in cow and buffalo milk [50], while in some cases slightly lower levels of TRP were reported in goat milk compared to other animal species [50,51]. Furthermore, the nutrient composition of yoghurts has been shown to be highly dependent on the technological process [45].

The typical recommended daily intake for tryptophan has been set by FAO/WHO at 4 mg/kg of body weight per day for adults [52], that is to say, 280 mg/day for a 70 kg adult [3].

According to the data reported in this study, three daily recommended servings of cow yoghurt (125 grams for a serving [53]) supply almost 60.6% of the recommended TRP daily intake for adults, while 84.3%, 48.8% and 70.9% of the recommended TRP daily intake is supplied by three servings of ewe, goat and buffalo yoghurt, respectively. It is worth noting that only two Greek yoghurt servings are enough for achieving the entire TRP recommended daily intake (108.5%), thanks to its high protein content.

TRP levels in yoghurts are generally of the same order of magnitude in milk [6,54], so a yoghurt serving (with the exception of Greek yoghurt, due to its typical production process) provides almost the same TRP intake of a milk serving. However, yoghurt’s shelf life is longer than milk’s, and yoghurt can also be consumed by lactose-intolerant people. Furthermore, according to the study of Bertazzo et al. [47], the TRP content in yoghurt does not decrease during the storage, so the TRP intake is guaranteed until the expiration date of the yoghurt. For all these reasons, yoghurt can be considered a good source of TRP, and its consumption should be encouraged, not only for ensuring the recommended daily intake of TRP, but also for its overall nutritive value, above all the probiotic properties and the calcium intake.

## 4. Materials and Methods 

### 4.1. Samples Preparation

In total, 22 commercial brands of yoghurt from different species were purchased in the local markets:

- 12 different brands of whole cow yoghurt;
- 2 different brands of whole ewe yoghurt;
- 2 different brands of whole goat yoghurt;
- 2 different brands of whole buffalo yoghurt;
- 2 different brands of whole cow yoghurt, lactose-free;
- 2 different brands of whole cow Greek yoghurt.

All samples were stored at 4 °C, as indicated on the label, prior to testing.

Tryptophan (TRP) was extracted by alkaline hydrolysis according to the method of Steven and Jorg [18]. In brief, 8 mL of NaOH 4.2 M was added to 0.5 g of the yoghurt samples. An appropriate amount of the internal standard (5-methyl-l-tryptophan) was added, then the oxygen was removed to avoid the oxidative degradation of TRP. The hydrolysis was carried out at 110 °C for 20 h. Afterwards, the samples were cooled in an ice bath and neutralized with HCl, added with EtOH and filled to the mark with phosphate buffer 0.2 M. The samples were filtered (0.2 µm) prior to the UHPLC analysis.

All the 22 commercial brands of yoghurt were analyzed in triplicate.

### 4.2. Chemicals

l-tryptophan (TRP) and 5-methyl-triptophan (M-TRP) were purchased from Sigma (Sigma-Aldrich Co., St. Louis, MO, USA).

The hydrochloric acid, sodium hydroxide and acetonitrile of HPLC grade were from Merck (Darmstadt, Germany). All the other chemicals used were of analytical purity. All solvents were filtered through 0.2 μm membrane filters (Phenomenex Inc., Torrance, CA, USA).

### 4.3. UHPLC Equipment and Conditions

A Nexera UHPLC system (Shimadzu Corporation, Kyoto, Japan), equipped with two LC-30AD pumps, an RF-20A fluorimetric detector and an SIL 30-AC autosampler, was employed for the chromatographic analyses. A Shim-Pak ODS II column (2.2 µm; 75 mm × 2 mm i.d., Shimadzu Corporation, Kyoto, Japan), maintained at 25 °C during the analysis, was used for the separation. The selected elution system, for a total of running time equal to 8.0 min, is reported in Table 5. The flow rate was set at 0.4 mL/min, while the injection volume was set at 1 µL. Tryptophan and 5-methyl-tryptophan were detected at 280 and 360 nm for excitation and emission wavelengths.

### 4.4. Method Validation

Standard stock solution of TRP (21.2 mg in 25 mL of deionized water) was prepared. Individual working standard solutions were prepared at six different levels by dilution in deionized water of the standard stock solution (0.085 µg/mL 0.170 µg/mL; 0.424 µg/mL; 0.553 µg/mL; 0.,848 µg/mL; 1.105 µg/mL).

Quantities of 100 µL of the internal standard 5-methyl-l-tryptophan (856 µg/mL in NaOH 0.05 M) were added before hydrolyzing all the samples.

The method’s performance parameters were evaluated according to the EURACHEM Guide 2014 [27]. In more detail, the method precision was evaluated through the measurements of repeatability (intra-day precision) and reproducibility (inter-day precision) upon direct injection of TRP standard solutions at six levels, respectively, on three replicates and on three non-consecutive days. The repeatability and reproducibility on yogurt samples at three levels, respectively, on three replicates and on three non-consecutive days, were considered as well to evaluate the variation due to the entire analytical procedure. Precision was expressed as relative standard deviation (RSD %). 

The limit of detection (LOD) and limit of quantification (LOQ) for TRP were calculated according to the following equations: LOD = X_b_ + 3 × SD_b_, LOQ = X_b_ + 10 × SD_b_, where X_b_ was the blank mean value (*n* = 10) and SD_b_ the blank standard deviation [27]. Procedural blanks [55], where deionized water was used in place of the yoghurt matrix, were used for determining LOD and LOQ.

The recovery was calculated by means of spiked samples, and expressed as relative spike recovery, according to the following equation: R (%) = [(x′ – x)/x_spike_] × 100. Here, x′ is the mean value of the spiked sample, x is the mean value of the non-spiked sample and x_spike_ is the added concentration [27].

### 4.5. Statistics

All the analyses were performed in triplicate. Data were reported as mean value with standard deviation (SD). Mean values were subjected to one-way analysis of variance (ANOVA), coupled with the Tukey’s post hoc test. Statistical analysis was performed using the PAST Software (2.17c version) [56].

## Figures and Tables

**Table 1:** Accuracy of the method expressed as Recovery (%) in yoghurt samples.

Spiked Levels	TRP Addition (µg/mL)	Recovery (%)
1	0.061	97.36 ± 0.70
2	0.091	98.90 ± 1.58
3	0.122	92.79 ± 1.09
4	0.152	100.12 ± 1.16

**Table 2:** Repeatability and reproducibility performances on pure standards.

std TRP (µg/mL)	Intra Day			Inter Day
	1 Day RSD%	2 Day RSD%	3 Day RSD%	RSD%
1.105	1.23	1.00	0.99	1.08
0.848	1.68	1.06	1.09	1.28
0.424	1.66	1.33	1.21	1.40
0.553	1.50	2.00	1.44	1.65
0.170	1.66	0.84	1.37	1.29
0.085	1.13	1.11	1.02	1.09

**Table 3:** Repeatability and reproducibility performances on yoghurt samples.

TRP Level (mg/100 g)	Intra Day			Inter Day
	1 Day RSD%	2 Day RSD%	3 Day RSD%	RSD%
121.97	2.59	2.87	1.79	2.59
62.96	1.11	1.14	1.18	1.71
35.19	1.36	1.55	1.27	1.76

**Table 4:** TRP contents (mg/100g) in commercial yoghurts from milk of different animal species.

Yoghurt	Sample Number	Mean	SD
Cow yoghurt (whole milk plain)	1.	41.94	0.37
	2.	41.28	0.08
	3.	41.44	0.20
	4.	41.70	0.60
	5.	40.78	0.26
	6.	42.00	0.13
	7.	45.48	0.12
	8.	44.36	1.03
	9.	48.82	0.38
	10.	49.22	0.17
	11.	48.91	0.06
	12.	49.31	0.14
Ewe yoghurt (whole milk plain)	1.	62.91	1.20
	2.	62.96	1.45
Goat yoghurt (whole milk plain)	1.	35.19	1.18
	2.	37.61	1.33
Buffalo yoghurt (whole milk plain)	1.	53.01	1.23
	2.	52.87	0.95
Cow yoghurt (lactose-free whole milk plain)	1.	47.93	0.47
	2.	50.36	0.39
Cow yoghurt (Greek-type whole milk plain)	1.	121.97	2.69
	2.	120.98	2.24

**Table 5:** Gradient elution system.

Time (Min)	CH_3_CN %	H_2_O %	mL/min
0.0	10	90	0.4
3.0	80	20	0.4
4.0	10	90	0.6
7.9	10	90	0.6
8.0	10	90	0.4
